# History and Evolution of the Otoscope

**DOI:** 10.7759/cureus.78101

**Published:** 2025-01-27

**Authors:** Cody Suh, Daniel Z Zhao, Latha Ganti

**Affiliations:** 1 Biology, The Lawrenceville School, New Jersey, USA; 2 Biology, Brown University, Providence, USA; 3 Emergency Medicine and Neurology, University of Central Florida, Orlando, USA; 4 Research, Orlando College of Osteopathic Medicine, Winter Garden, USA; 5 Medical Science, The Warren Alpert Medical School of Brown University, Providence, USA

**Keywords:** guy de chauliac, history of medicine, medical instrument, otolaryngology, otoscope

## Abstract

This paper provides an in-depth review of the evolution of the otoscope in the medical field, examining its development through a historical lens. The otoscope has significantly revolutionized the field of otology, becoming an essential tool used globally by healthcare practitioners to identify ear, nose, and throat (ENT) disorders. It is commonly used to inspect the auditory canal for conditions such as cerumen impaction and acute otitis media. From its early conception focused on studying the middle ear to its present-day form, this paper explores the otoscope's rich history, highlighting key pioneers in its development and discussing its future implications in clinical practice.

## Introduction and background

**Figure 1:**
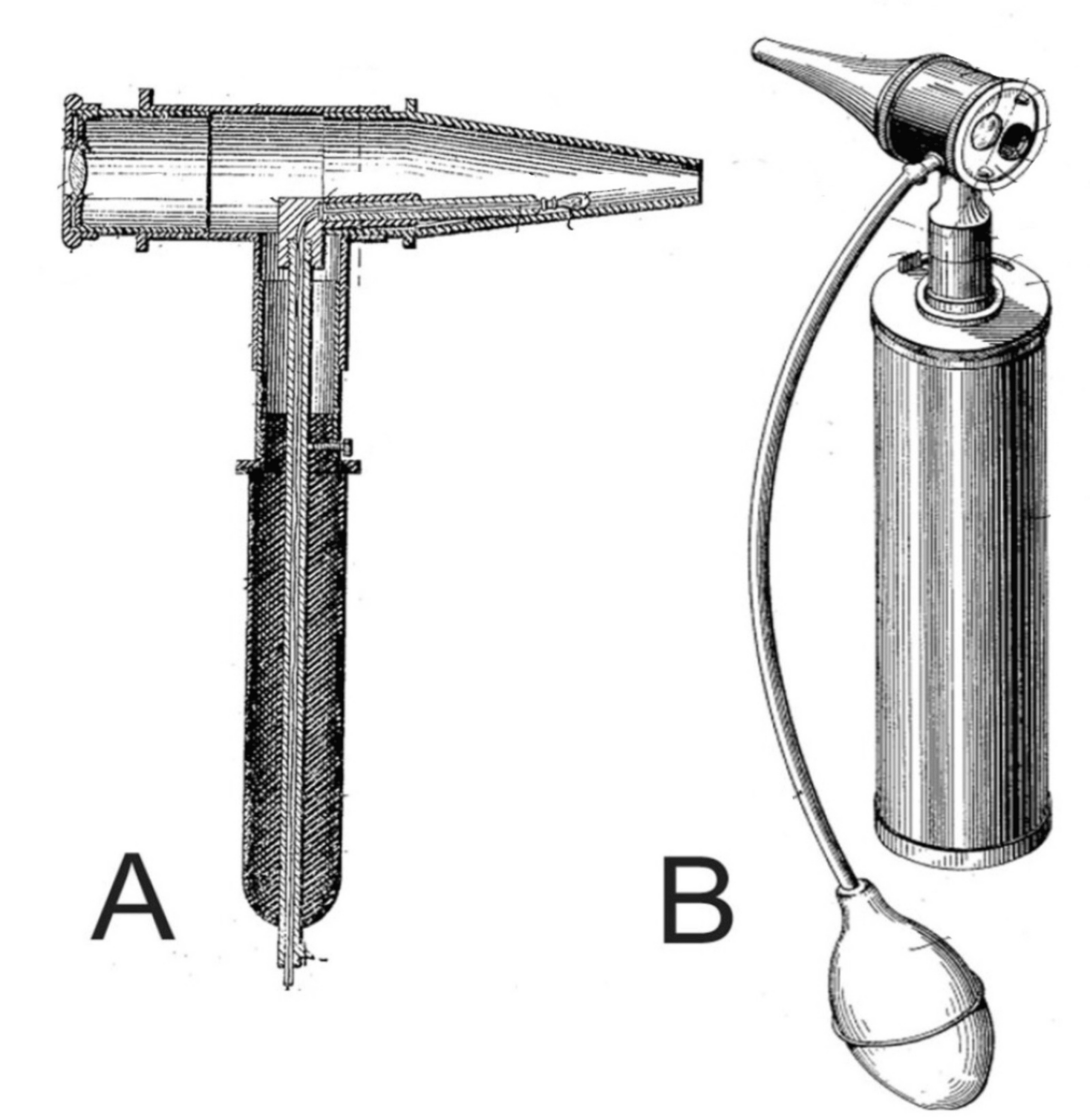
Visual diagram of a pneumatic otoscope (A) alongside a modern-day otoscope (B) Source: [1].

Ear examinations are a standard component of annual physical exams. The otoscope remains the gold-standard medical device for these examinations, a specialized tool designed to illuminate and magnify the auditory canal and tympanic membrane for accurate diagnosis (Figure 1).

**Figure 2:**
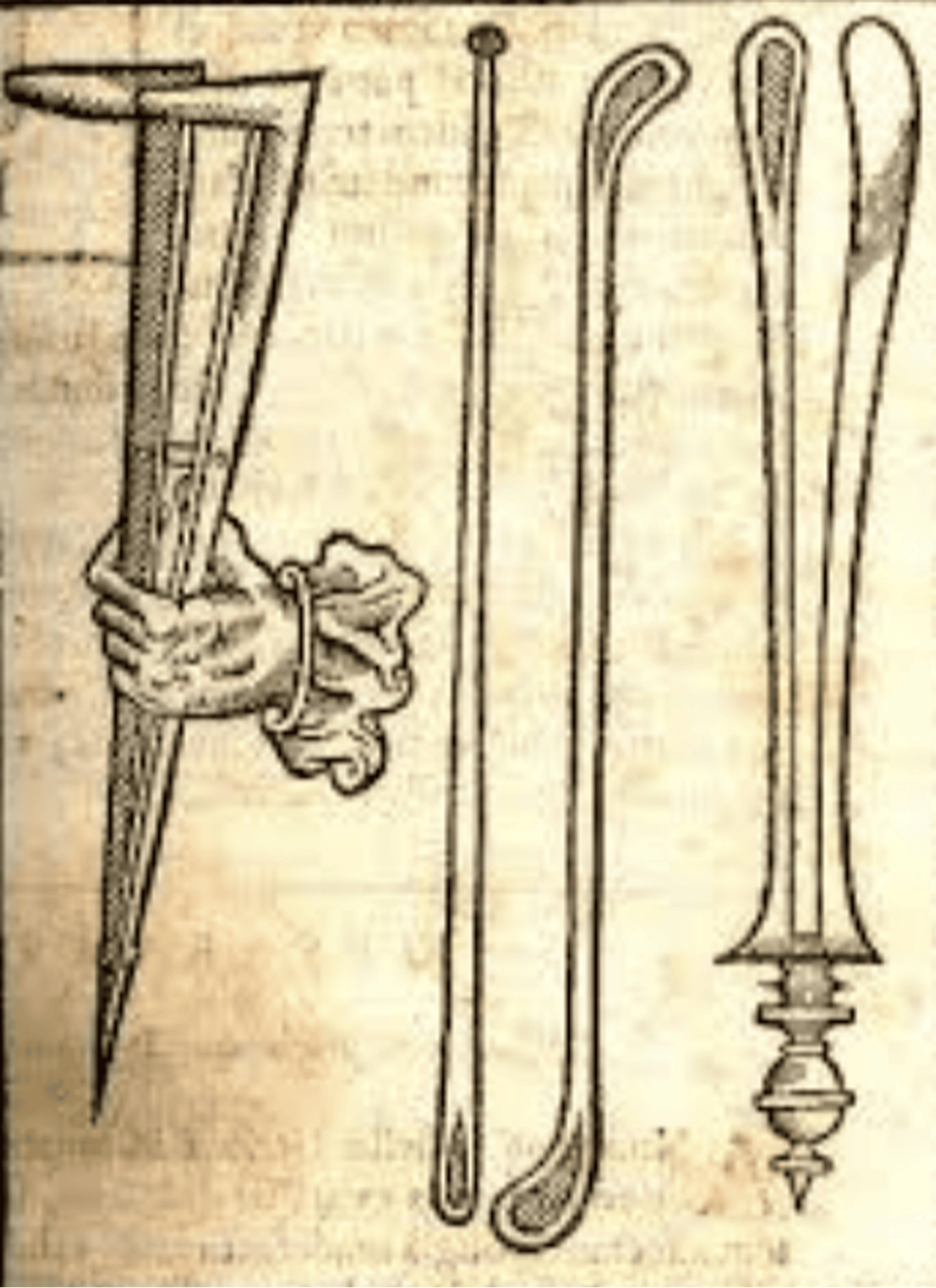
Photograph of Fabricus Hildanus’ designs of the primitive otoscope Source: [9].

The otoscope provides a clear, magnified, and illuminated view of the auditory canal, allowing otolaryngologists to assess for conditions such as cerumen impaction, abnormalities, and infections in the auditory canal and tympanic membrane [1-3]. Numerous distinct designs and prototypes of the otoscope have been developed over time, but the universally standardized model used by otolaryngologists today features a cone-shaped ear speculum attached to a metal handle containing a light source and batteries [4]. Due to the auditory canal’s small size and delicate structure, direct observation with the naked eye is insufficient for diagnosing ear, nose, and throat (ENT) disorders, which often require specialized tools for accurate evaluation. Common ENT disorders such as otitis media and earwax impaction can lead to hearing loss, pain, and infections, underscoring the critical role of the otoscope in effective diagnosis and management. Before the invention of the otoscope, no practitioner had attempted to use light to examine body cavities, limiting diagnostic accuracy for ear conditions. This often led to missed diagnoses due to inadequate visualization of the auditory canal and tympanic membrane [5]. In 1585, physician Tulio Caesare Aranzi revolutionized the use of lighting for ear examinations. Over the next four centuries, many physicians refined and advanced this method. One such example is Archibald Cleveland, an English surgeon, who used a candle and a biconvex lens to illuminate body cavities, complementing the otoscope technology of the 18th century [5]. The first true otoscope was developed in 1363 by French physician Guy de Chauliac [6]. This concept was later refined by Fabricus Hildanus in 1646 and German otologist Wilhelm Kramer in the 19th century, who developed a prototype for clearer imaging [6]. In 1846, Lincke and Schmalz introduced the funnel-shaped speculum, forming the foundation for modern otoscopes [5]. The 20th century brought further advancements, including the shift from metal to plastic speculums and the introduction of improved light sources and fiber optics. These innovations revolutionized the field of otology, providing clearer images of the ear and allowing practitioners to identify abnormalities that were previously difficult to detect. The development of the otoscope paved the way for other diagnostic tools, such as rhinoscopy and ophthalmoscopy. Even today, otoscopes continue to evolve, with modern versions enabling practitioners to conduct exams through smartphone screens when connected to electronic devices [7,8]. The history and development of the otoscope have significantly impacted the medical field and produced an instrument used universally across healthcare systems (Figure 2).

## Review

Introduction to the otoscope

The otoscope, consisting of the modern-day ear speculum and a metal rod, revolutionized the field of otoscopy. However, this invention underwent centuries of advancements and prototypes before achieving the universally standardized design used by practitioners worldwide today. Prior to the otoscope, specialists relied on candlelight and speculums to see inside the ear [4-6]. Speculums are medical instruments that help practitioners view hollow regions of the body [3]. However, these early devices did not enable a comprehensive ear examination, as they did not allow for direct visualization of the tympanic membrane [5].

Historical evolution of the otoscope

In 1363, in an effort to develop a more effective and accurate method to examine the ear, physician Guy de Chauliac proposed the earliest version of the ear speculum [5]. The initial design was tong-like, creating a pathway for early versions of speculums in the field of otology [6]. Unlike modern speculums, Guy de Chauliac’s design lacked the funnel shape and magnification capabilities, as technology at the time had not yet advanced sufficiently [6]. In 1646, Fabricus Hildanus further improved Chauliac's prototype, creating a version of the instrument used by physicians of that era. For the proper use of this instrument, the speculum was fully inserted and closed within the ear, and the tongs would then open the ear for examination of its interior for abnormalities or disorders [6]. In 1836, Wilhelm Kramer developed the widely used speculum in Germany, while many different versions of the speculum emerged across Europe. One of the most notable designs came from Ignaz Gruber in Vienna, who created a conical-shaped speculum with a single rod, moving closer to the design used in modern otoscopes [6].

The first otoscope was devised by J. Bruton, an English surgeon, who combined an ear speculum, a tool used to widen the auditory canal for examination, with a perforated mirror and a magnifying glass. While this may be the first-ever recorded otoscope, the ear speculum most commonly seen today was not invented until 1881 by A. Hartman [6]. By this time, diagnosing diseases of the middle ear remained challenging, as this part of the ear had not yet been thoroughly studied [4]. German otologist Von Troeltsch discovered a method using natural light and a concave mirror to examine the tympanic membrane [4]. However, a few years later, the discovery of plastic and the miniaturization of the light bulb further promoted the use of otoscopes. Within a decade of Thomas Edison's invention of the light bulb, doctors no longer had to rely on natural light reflected off mirrors. The miniaturized light bulb efficiently illuminated the ear, enabling more precise examinations at any time of day [4]. This innovation also spurred the development of other medical instruments, such as the ophthalmoscope [2]. The material of the ear speculum transitioned from metal to plastic, as it was significantly cheaper and easily disposable after use [5,6]. By the early 20th century, electrically powered otoscopes were widely available.

**Figure 3:**
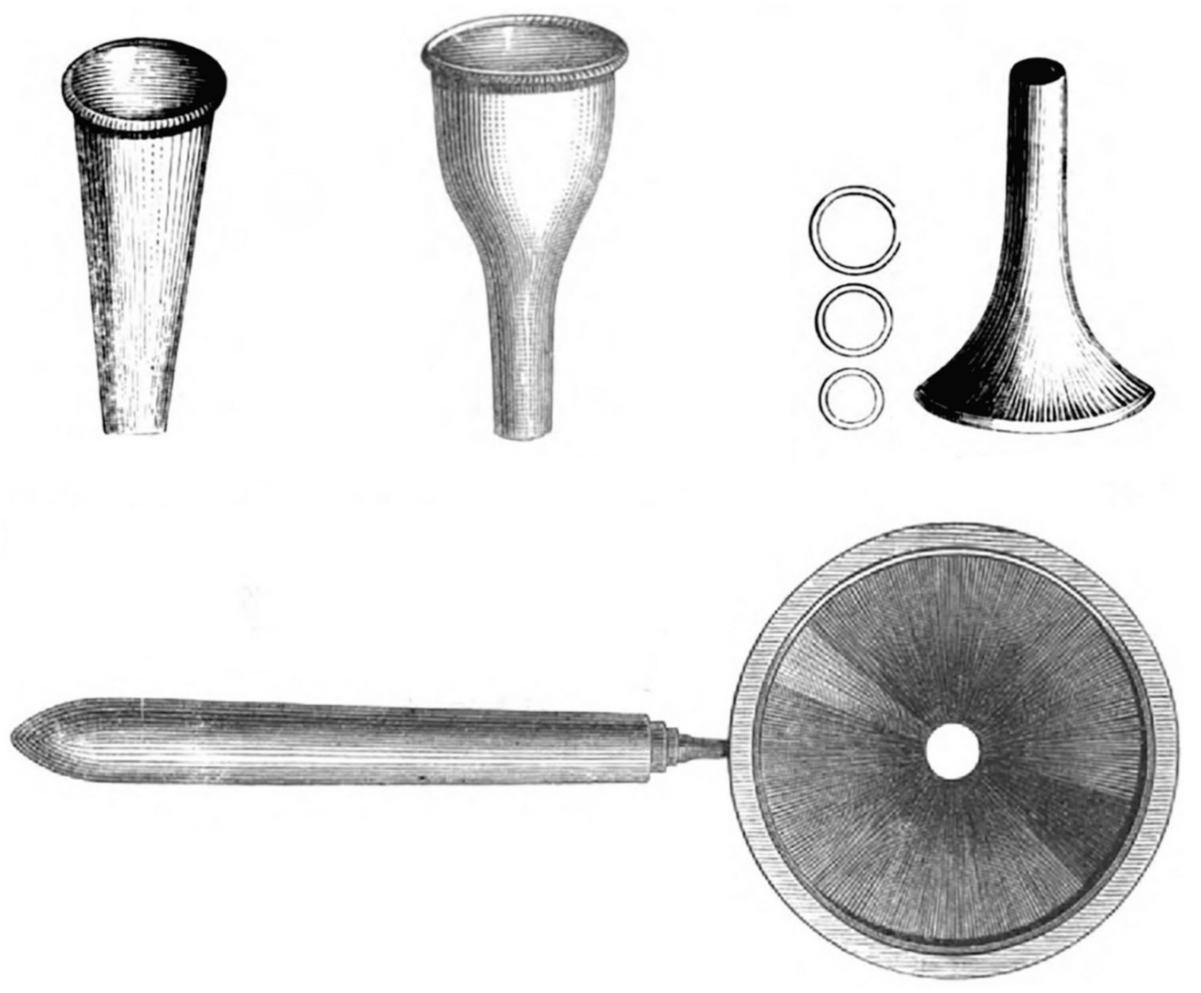
Visual representation of the evolution of the ear speculum and otoscope Source: [1].

Peter T. Geyerman developed the first fully compact otoscope powered by batteries [4]. Later in 1910, Henry L. De Zeng created the first pneumatic otoscope, which allowed for testing pressure changes in the ear and diagnosing conditions such as acute otitis media [10]. In modern practice, advancements in technology have enabled otoscopic examinations to be displayed directly on devices such as smartphones, television screens, and cameras [10]. During the SARS-CoV-2 pandemic, when in-person visits were limited, people began using their phones as otoscopes, conducting self-exams and sending images to their doctors. A study conducted by Dr. Donelly, Dr. Quaraishi, and Dr. McShane demonstrated that this new technology helped medical students more easily locate parts of the middle ear [7,8]. However, the technology was less effective with local healthcare workers, highlighting the need for a thorough education on ear anatomy [7,8]. Although this innovation was a major leap in the otoscope’s evolution, this version of the otoscope still has its accessibility flaws. In many rural areas, people may lack access to smartphones or the Internet, limiting the reach of this technology. Other modern improvements include the integration of fiber optics to direct the light source more precisely to the required area. Additionally, new techniques, such as using vibrations and lasers to assess middle ear function, have further advanced otoscopic practice [10]. Over the years, the ear speculum and otoscope have undergone continuous innovation and technological improvements, enhancing their functionality and impact in the medical field (Figure 3).

Despite the usefulness of the otoscope, accurately diagnosing ear diseases remains challenging, as many ear conditions are subjectively reported by the patient [4]. Also, one-quarter of adults and half of pediatric consultations are related to ENT issues [2]. In emergency departments, two-thirds of patients present with ENT-related concerns [2]. Given the frequency with which otoscopes are used in patient care, it is crucial that otolaryngologists and all healthcare providers are proficient in their use, as the inability to utilize an otoscope can negatively impact a patient if an insufficient ear exam is performed. With limited exposure to otolaryngology, many medical students lack the knowledge or confidence to perform a proper ear exam [2]. This gap in education can lead to inefficient examinations and, ultimately, poor patient outcomes.

Technological milestones and innovations

Otoscope technology continues to evolve with the integration of digital imaging, artificial intelligence (AI), and enhanced connectivity. The increasing prevalence of digital otoscopes equipped with high-resolution imaging capabilities allows for a clearer visualization of the auditory canal and tympanic membrane. This improvement aids in more accurate diagnoses and enhances telemedicine applications by allowing remote assessments [11]. The adoption of video-otoscopy in remote otological assessments has significantly improved healthcare outcomes in various regions. A systematic review published in 2021 found that nonspecialist facilitators could successfully capture diagnostic-quality images, with a pooled failure rate of 26%, demonstrating the potential of video-otoscopy to enhance otological care in underserved areas [11]. This innovation reduces diagnostic variability, promotes consistency among practitioners, and fosters greater reliability in the management of ear health.

The integration of AI into otoscopy is an emerging advancement in medicine. Systems like OtoPhoto (Johns Hopkins Medicine, Baltimore, MD, USA) utilize AI to improve diagnostic methods by addressing user errors and offering detailed analyses of ear conditions [12]. Similarly, the HearScope (HearX Group, Pretoria, South Africa) system utilizes AI-powered diagnostic support, enhancing the efficiency and accessibility of diagnosing ear diseases [13]. These technological innovations promote consistent diagnoses and minimize variability across practitioners, fostering greater reliability in otological care. The integration of AI-powered systems in otoscopy has the potential to revolutionize otological care by standardizing diagnostics, reducing practitioner variability, and improving the accuracy and accessibility of ear disease detection.

The use of affordable digital otoscopes that connect to smartphones is on the rise, enabling patients to conduct self-examinations and share captured images directly with healthcare providers. A study demonstrated that 95% of ear images obtained by patients using digital otoscopes were of acceptable quality, indicating the feasibility of such approaches in telehealth [14]. Smartphones enhance otological care by serving as accessible, cost-effective tools for capturing high-quality otoscopic images, enabling improved diagnostics, and facilitating telemedicine in underserved regions [15]. 

## Conclusions

The creation and evolution of the otoscope have been pivotal to the field of otology. From the early use of candles for ear examination to the development of modern-day otoscopes, its progress has played a critical role in detecting ear disorders, identifying abnormalities, and studying the anatomy of the middle ear. Advancements in other fields, such as the invention of lightbulbs and their application in the ophthalmoscope, provided significant inspiration for otoscope innovators. This inspiration became foundational in the otoscope's design, particularly through the integration of a light source and mirror. Over the centuries, these developments have had a profound impact on global health, and ongoing innovation remains essential for advancing healthcare and benefiting society at large. Historical advancements in otoscopy include the transition from basic specula to modern otoscopes equipped with magnification and illumination. Recent innovations, such as AI-powered and digital otoscopy systems, have further enhanced diagnostic accuracy and accessibility. The future of otoscopy holds immense potential, with AI and digital systems poised to standardize care, improve early detection of ear disorders, and expand access to underserved regions.
